# Pneumosinus Dilatans of the Frontal and Ethmoidal Sinuses Revealed by Conscience Disorder and Respiratory Distress: Case Report and Literature Review

**DOI:** 10.7759/cureus.53539

**Published:** 2024-02-04

**Authors:** Bouchra Chahboun, Ousmane Kaba, Ghizlane El Aidouni, Houssam Bkiyar, Brahim Housni

**Affiliations:** 1 Intensive Care Unit, Faculty of Medicine and Pharmacy of Oujda, Mohammed First University, Oujda, MAR; 2 Intensive Care Unit, Mohammed VI University Hospital, Oujda, MAR; 3 Anesthesiology and Intensive Care Unit, Mohammed Vi University Hospital, Oujda, MAR

**Keywords:** conscience disorder, subfalcine herniation, ischemic stroke, ethmoidal sinus, frontal sinus, pneumosinus dilatans

## Abstract

An abnormal enlargement of the air-filled paranasal sinuses is referred to as pneumosinus dilatans. Typically discovered incidentally through radiological examinations, it infrequently manifests as cosmetic, neurological, ocular, or rhinological pathologies. Thorough evaluation for associated conditions is essential in patients with pneumosinus dilatans, including meningiomas of the anterior skull base or the optic nerve sheath. In our work, we report a 75-year-old female patient who presented with dysarthria and lower facial asymmetry. The computed tomography (CT) scan revealed pneumosinus dilatans of the frontal and ethmoidal sinuses with subfalcine herniation. During hospitalization, the patient presented with conscience disorder secondary to ischemic stroke and respiratory distress secondary to aspiration pneumonia. In our work, we also discuss reported cases of the English literature.

## Introduction

Pneumosinus dilatans (PSD) is an uncommon and benign condition affecting the paranasal sinuses. It involves the abnormal expansion of a paranasal sinus caused by hyperpneumatization [1-3]. The term “pneumosinus dilatans” was first used in 1918 [4]. Distinguishing PSD from hypersinus, where the sinus is hyperaerated and enlarged but stays within the normal bony boundaries, and from pneumocele, where there is thinning of the bony sinus walls as well, is crucial [5]. The highest incidence occurs in the frontal sinus (63%), followed by the sphenoidal sinus (25%), maxillary sinus (19%), and ethmoidal sinus (18%). Typically, it affects a solitary sinus cavity, although there are instances where multiple or all sinuses may be involved [6]. The presentation of this condition varies widely, ranging from asymptomatic individuals to those experiencing symptoms such as nasal obstruction, facial deformities, pain, or visual changes.

## Case presentation

**Figure 1:**
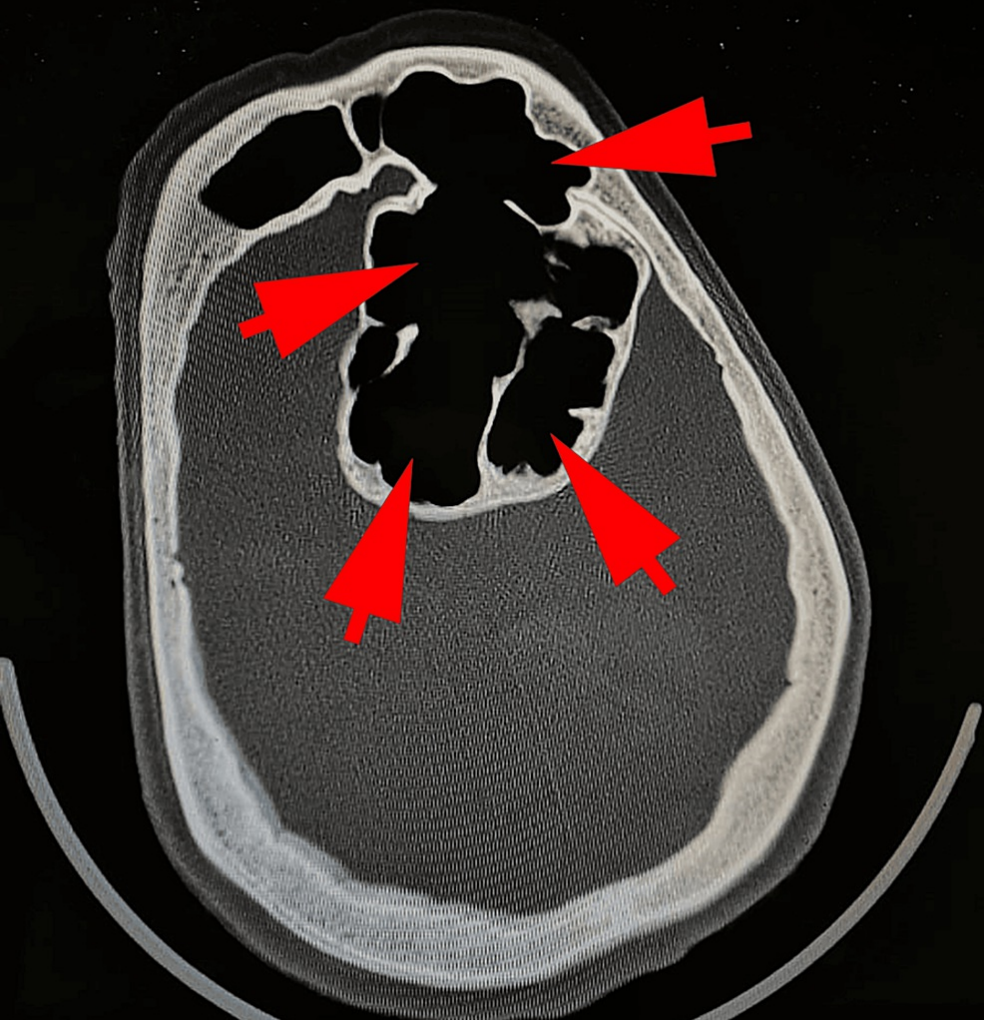
The computed tomography (CT) scan revealed significant enlargement of the frontal and ethmoidal paranasal sinuses (arrows), characterized by hyperpneumatization, consistent with PSD

We report a 75-year-old female patient who presented with acute onset of dysarthria and lower facial asymmetry. She has a history of uterine leiomyosarcoma for which a hysterectomy and chemotherapy were performed one year before the current admission, with follow-up revealing no signs of recurrence. No history of trauma, nasal bleeding, facial pain, headache, nasal obstruction, or any ocular complaints have been reported by the patient. Clinical examination has confirmed dysarthria and lower facial asymmetry, with a Glasgow score at 15, a high arterial blood pressure at 170/110 mmHg, and a normal oxygen saturation at 96% without oxygen supply. Visual acuity was normal, and pupils were reactive. The computed tomography (CT)-scan revealed significant enlargement of the frontal and ethmoidal paranasal sinuses, characterized by hyperpneumatization with the frontal sinus reaching 3cm in greatest dimension and the ethmoidal sinus measuring 6.5cm in greatest dimension, consistent with PSD. The bony thickness was remarkably reduced in the areas of pneumosinus (Figure 1).

**Figure 2:**
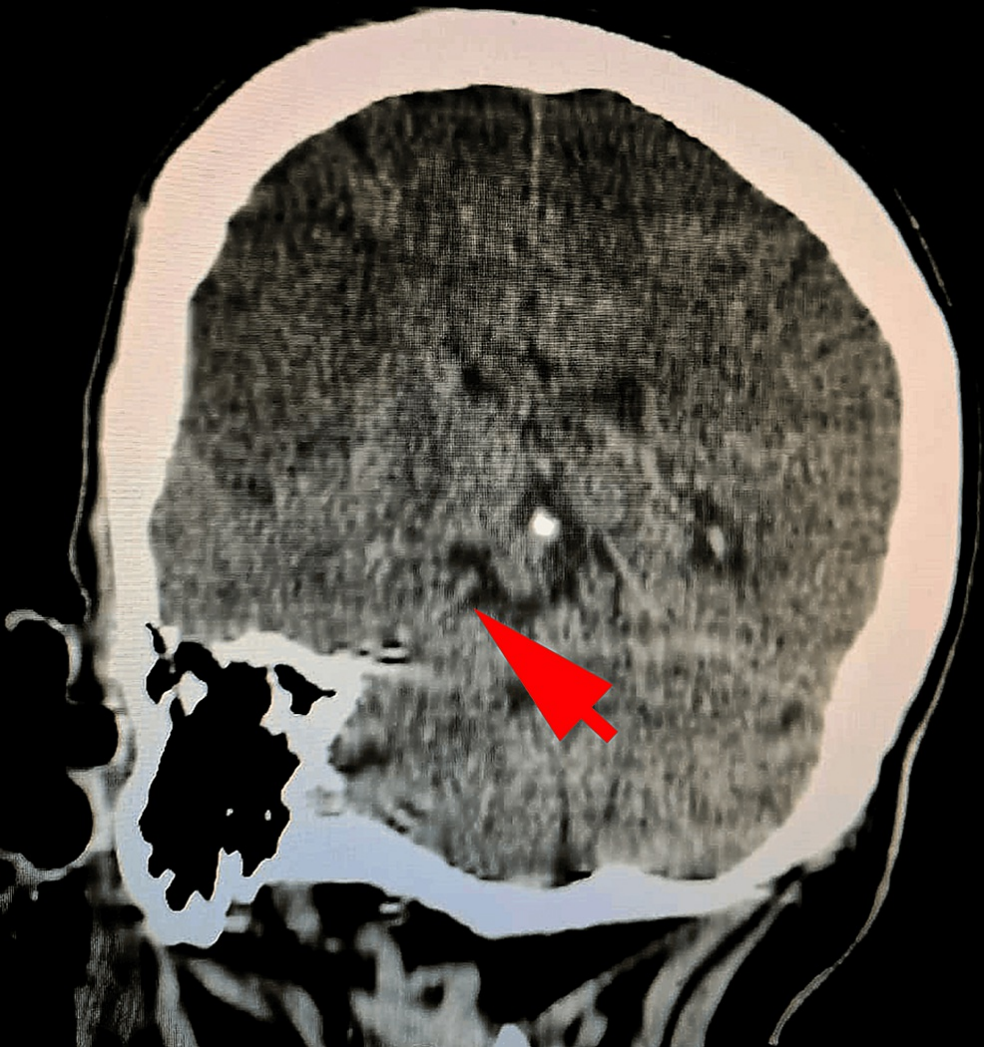
The computed tomography (CT) scan revealed the presence of subfalcine herniation (arrow)

A subfalcine herniation was also revealed by the CT scan (Figure 2).

**Figure 3:**
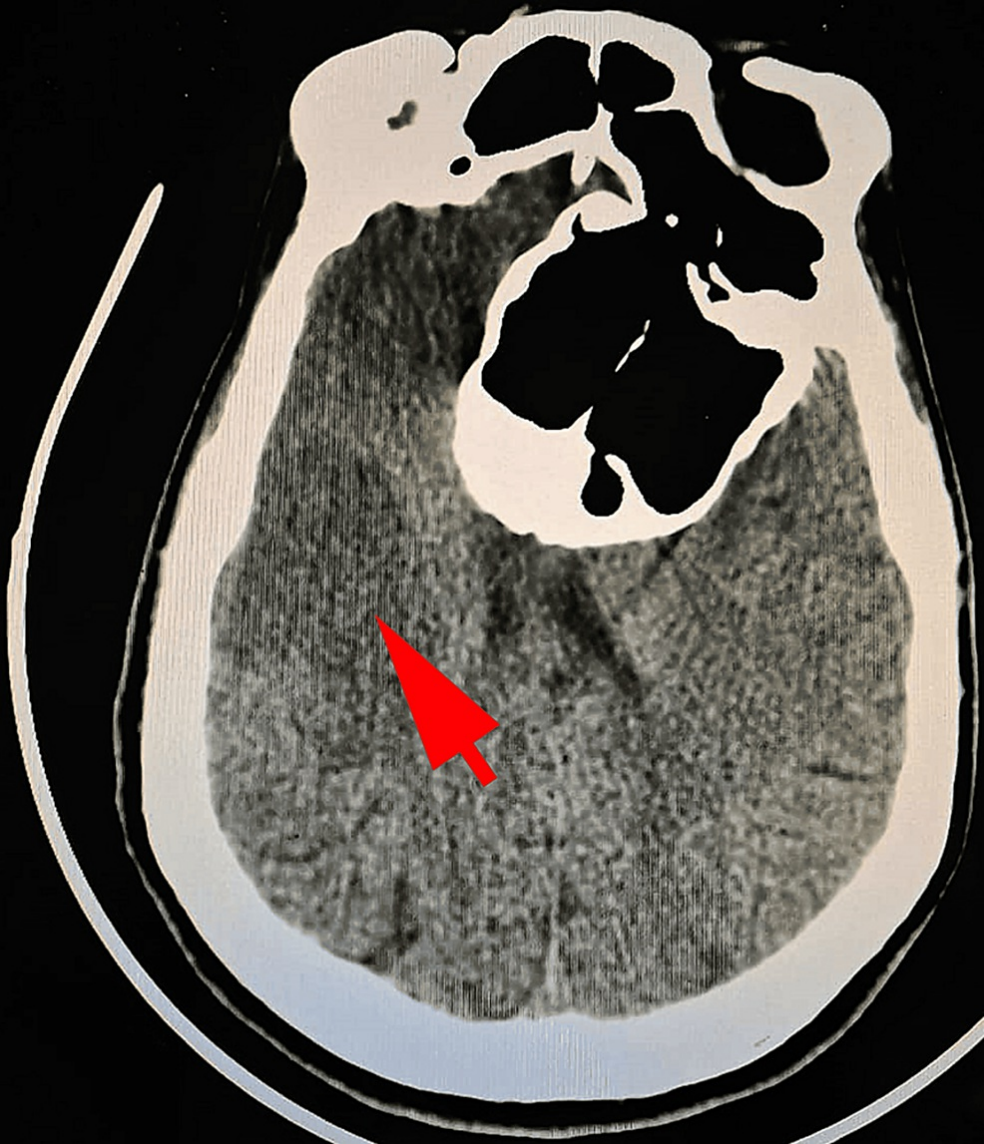
The computed tomography (CT) scan revealed spontaneous hypodensity (arrow) on right cerebral medial artery with loss of grey-white matter differentiation and gyral effacement

Initial management of the patient was based on anti-hypertensive therapy using amlodipine. During hospitalization, the patient presented disorder of conscience with deterioration of the of Glasgow Coma Scale score to 8. Physical examination revealed normal temperature at 37.5 °C, a normal arterial blood pressure at 127/67 mmHg, a normal mean arterial pressure at 88mmHg. The heartbeat was normal at 93 bpm. The respiratory rate was normal at 16 breaths per minute, and the oxygen saturation was low at 80% without oxygen supply and at 93% after 6 liters/min oxygen therapy. A bilateral miosis was observed. Laboratory explorations revealed normal glycemia at 1,19 g/L, and normal sodium, potassium, and calcium levels. Arterial blood pH, PO2, and PCO2 have normal values at 7.40, 98 and 39.5mmHg, respectively. A biological inflammatory syndrome was found, with elevation of the C-reactive protein at 47mg/L and erythrocyte sedimentation rate (ESR), at 40mm/h. A CT scan was performed and revealed the presence of a de novo ischemic stroke on the territory of the right middle cerebral artery (Figure 3).

The thoracic CT scan revealed a consolidation on the upper right pulmonary lobe that was associated to a mild right pleural effusion. The management of the patient was based on oxygen therapy, antibiotic therapy with ceftriaxone at 2g per day, and thromboprophylaxis with enoxaparin at 4,000 IU/12h.

## Discussion

The rare condition known as PSD typically occurs in young men aged between their 20s and 40s; however, it can impact individuals of both genders at any stage of life [7]. This is what can be observed in our reported case who is a 75-year-old female patient. Despite the recording of a significant number of cases, the true incidence and etiology of the condition remain unclear and given that a substantial proportion of patients either solely express cosmetic concerns or receive a diagnosis based on radiological findings, it is reasonable to speculate that the actual incidence might surpass initial expectations. A bimodal age distribution, with peaks observed at ages 16 to 25 and 36 to 45 years has been also reported, indicating potential variations in pathophysiology across different age groups [6]. Paranasal sinus dilatation is a rare disorder of uncertain origin, marked by abnormal hyperaeration and enlargement of one or more paranasal sinuses [7]. The expansion may encompass either a specific section of the sinus or the entire sinus itself [8].

A classification system was suggested by Urken et al. [5], categorizing these expanded and air-filled sinuses into three groups, specifically: Hypersinus, is typically asymptomatic, and surpasses the upper limit of the sinus's normal boundaries but remains within the standard range of the affected bone, exhibiting normal sinus walls; pneumatocoele which extends past the normal anatomic confines of the sinus, with local pressure symptoms being sometimes evident, and PSD, extending beyond the usual anatomic boundaries of both the sinus and the affected bone. PSD features displaced sinus walls and maintains normal bony thickness. Clinical presentation may include localized pressure symptoms.

Several hypotheses have been proposed for primary PSD, including hormonal imbalance, infection with gas-forming organisms, and the spontaneous discharge of mucocele [9]. A widely accepted theory involves a one-way (ball) valve effect, supported by the presence of polypoid mucus in the drainage pathway of the affected sinus [10]. Jankowsky et al. [11] suggested osteogenic diseases as a potential cause. The observed connection between PSD and meningiomas and arachnoid cysts, both intracranial pathologies, has given rise to a hypothesis suggesting that disruptions in cerebrospinal fluid (CSF) dynamics may lead to the dilation of paranasal sinuses through alterations in intracranial pressure [9,12]. Arachnoid cysts and meningiomas can interfere with CSF dynamics by compressing cerebral blood flow. This compression of cerebral arteries may impede CSF flow within the ventricular system, while compression of cerebral veins can hinder CSF absorption. Moreover, there is a belief that pathological enlargement of sinuses in PSD patients might be triggered by hormonal changes during puberty in genetically predisposed individuals, as some experience disease onset or rapid progression during this period [7]. PSD exhibits a bimodal age distribution, leading some authors to propose that distinct pathophysiologic processes may prevail in different age groups. This could involve a hormonal process in younger patients and a ball-valve obstruction in older patients [7].

In a 2021 literature review, about 90 articles related to PSD, it has been mentioned that a total of 171 cases of PSD were described from 1918 to 2021. Among the 171 patients, 69.5% were males. 31.5% of the patients and fell within the 11-20 years age bracket [13]. More prevalent than multi-sinus involvement (as in our reported case with involvement of frontal and ethmoidal sinuses), was the occurrence of individual sinus involvement (80.1%), with the frontal sinus being the most commonly affected and the ethmoid sinus the least. The predominant presentation was swelling over the forehead. Cosmetic concerns were the sole presentation in 19.3% of the patients. Among the cases, 85 (49.7%) were associated with a concurrent pathology, with meningioma and arachnoid cyst being the most frequently observed. To our knowledge, we report the first case of PSD that was revealed by dysarthria and that was complicated by ischemic stroke and conscience disorder [13]. Among the 171 reported patients with PSD, 74 had symptoms related to vision, including diplopia, decreased vision, or complete loss of vision, were reported [13].

Many reports suggest possible associations to different pathologies: Desai et al. determined that sphenoid PSD carried a 24% probability, and frontal PSD had a 22% likelihood of being associated with either meningioma or arachnoid cyst. For those with ethmoidal involvement, there was a 17% incidence of arachnoid cyst [12].

Typically, patients with PSD are asymptomatic; however, if symptoms are present, they vary based on the affected sinus. Clinical manifestations of PSD encompass diverse facial deformities, such as proptosis or exophthalmos, cheek mass, alveolar swelling, prominence of the nasolabial fold, nasal obstruction, and visual acuity impairment. Frontal bossing is the most frequently observed presentation in cases involving the frontal sinus. Sphenoidal and ethmoidal PSD have been associated with ocular symptoms ranging from blurred vision to temporal field defects or sudden loss of vision. In instances of sphenoid sinus involvement, 52% of PSD patients report vision loss, and 22% of those with ethmoidal PSD exhibit exophthalmos [13].

The engagement of the maxillary sinus is also linked to cosmetic deformities. It is crucial to differentiate maxillary PSD from other maxillary sinus pathologies, such as neoplastic lesions, mucoceles, and complicated acute rhinosinusitis [14]. The correlation between PSD and fibro-osseous lesions and other osteogenic disorders may be attributed to abnormal bone formation. Given its association with syndromic diseases, exploring a genetic connection in these patients may be prudent. To our knowledge, we report the first case of PSD that was revealed by dysarthria and that was complicated by ischemic stroke and conscience disorder [13].

PSD patients should receive an ophthalmologic examination to detect signs of optic neuropathy, particularly in cases related to sphenoid or ethmoid sinuses [13]. In addition to visual acuity, pupillary reflexes, and color vision, it is advisable to perform thorough assessments of oculomotor function and facial sensation.

In the diagnosis of this condition, CT scans and MRI are pivotal as they provide information regarding bony deformities and erosions. They also help rule out any associated intracranial abnormalities [13]. On a coronal section of a CT scan, the width and height of the affected sinus can be measured in millimeters, while the volume can be quantified in cubic meters [13]. Radiological assessment is essential for the diagnosis of PSD, enabling the determination of extent, causality, and identification of associated conditions. In our case, it has enabled to reveal complications of PSD which were ischemic stroke and subfalcine herniation.

On the therapeutic level, patients diagnosed radiographically with asymptomatic PSD may undergo regular imaging to monitor disease progression [13]. For those expressing cosmetic concerns, the consideration of surgical recontouring of the affected sinus is warranted [15]. In instances of PSD affecting the frontal sinus, it is advisable to contemplate endoscopic evaluation of the nasofrontal duct to investigate potential ball-valve obstructions [3]. severe visual impairment may necessitate early surgical intervention. While there is limited supporting evidence for surgery, the presence of optic atrophy on radiology indicates that decompression would be the most appropriate course of action.

## Conclusions

The actual occurrence and cause of PSD remain uncertain, although there is an undeniable association with meningiomas, arachnoid cysts, and vision loss. Maintaining a high level of suspicion is crucial to recognize PSD as the underlying cause of declining vision, as reported in the literature, or in cases of conscience disorder. Prompt surgical intervention should be initiated.
